# Metabolic syndrome in Xinjiang Kazakhs and construction of a risk prediction model for cardiovascular disease risk

**DOI:** 10.1371/journal.pone.0202665

**Published:** 2018-09-06

**Authors:** Lei Mao, Jia He, Xiang Gao, Heng Guo, Kui Wang, Xianghui Zhang, Wenwen Yang, Jingyu Zhang, Shugang Li, Yunhua Hu, Lati Mu, Yizhong Yan, Jiaolong Ma, Yusong Ding, Mei Zhang, Jiaming Liu, Rulin Ma, Shuxia Guo

**Affiliations:** 1 Department of Public Health, Shihezi University School of Medicine, Shihezi, Xinjiang, China; 2 Department of Nutritional Sciences, The Pennsylvania State University, University Park, United States of America; 3 Department of Pathology and Key Laboratory of Xinjiang Endemic and Ethnic Diseases (Ministry of Education), Shihezi University School of Medicine, Shihezi, Xinjiang, China; The University of Texas at El Paso, UNITED STATES

## Abstract

**Background:**

The high prevalence of metabolic syndrome (MetS) and cardiovascular diseases (CVD) is observed among Kazakhs in Xinjiang. Because MetS may significantly predict the occurrence of CVD, the inclusion of CVD-related indicators in metabolic network may improve the predictive ability for a CVD-risk model for Kazakhs in Xinjiang.

**Methods:**

The study included 2,644 subjects who were followed for 5 years or longer. CVD cases were identified via medical records of the local hospitals from April 2016 to August 2017. Factor analysis was performed in 706 subjects (267 men and 439 women) with MetS to extract CVD-related potential factors from 18 biomarkers tested in a routine health check-up, served as a synthetic predictor (SP). We evaluated the predictive ability of the CVD-risk model using age and SP, logistic regression discrimination for internal validation (n = 384; men = 164, women = 220) and external validation (n = 219; men = 89, women = 130), calculated the probability of CVD for each participant, and receiver operating characteristic curves.

**Results:**

According to the diagnostic criteria of JIS, the prevalence of MetS in Kazakh was 30.9%. Seven potential factors with a similar pattern were obtained from men and women and comprised the CVD predictors. When predicting CVD in the internal validation, the area under the curve (AUC) were 0.857 (95%CI 0.807–0.898) for men and 0.852 (95%CI 0.809–0.889) for women, respectively. In the external validation, the AUC to predict CVD were 0.914 (95%CI 0.832–0.963) for men and 0.848 (95%CI 0.774–0.905) for women. It is suggested that SP might serve as a useful tool in identifying CVD with in Kazakhs, especially for Kazakhs men.

**Conclusions:**

Among **7** potential factors were extracted from 18 biomarkrs in Kazakhs with MetS, and SP may be used for CVD risk assessment.

## Introduction

A recent statistical report [1] indicates that China has 290 million people with cardiovascular disease (CVD), with a mortality rate higher than those for cancer and other diseases, accounting for more than 40% of all deaths. Moreover, the CVD mortality rate was higher in rural areas than in urban areas. The increased risk of CVD was due to the accumulation of risk factors such as obesity, dyslipidemia, hyperglycemia, and high blood pressure, and these risk factors are the major components of metabolic syndrome (MetS). Although the definition varies [2–6], MetS includes 4 primary components: hypertension, hyperlipidemia, central obesity, and impaired glucose tolerance. Each component interacts with other components to form a complex metabolic network [6]. The MetS metabolic network also consider serum liver enzyme levels [7,8], liver function index [9,10], and renal function index [11,12]. However, it remains unclear whether these indicators can be used as predictors in a CVD-risk model.

Many CVD risk assessment tools have been described, the most commonly cited one is the Framingham Study (FRS) model. Because of the different research populations, FSR could not be applied to all groups[13]. Subsequently, many studies have increased the predictive power of FRS for CVD by refining the study populations [14], increasing the number of risk factors [15], and using appropriate predictors [16]. Moreover, the CVD predictive model is constructed using the Whitehall integral model [17], neural network model [18], QRISK score [19], and components of MetS extracted by exploratory factor analysis (EFA) as CVD predictors [20]. These predictive models analyzed all aspects of CVD, and continuously supplemented the shortcomings of FRS prediction. However, the above CVD predictive models have local advantages and weak extrapolation ability [14]. Therefore, in order to effectively prevent and treat CVD, it is necessary to construct a targeted CVD prediction model.

Ethnic Kazakh is the most populous nomadic ethnic group in Xinjiang, China, and is mainly resides in mountain pasturelands in northern Xinjiang. Previous research found that the prevalence of MetS, hypertension, and obesity were higher in Kazakhs than in other ethnic groups in the same area [21–23]. Therefore, this study used EFA to identify potential factors associated with MetS in Kazakh patients. Latent factors have been used to build a predictive model of CVD risk for the Kazakh population in rural Xinjiang. The results provided the basis for the developing interventions for CVD and other chronic diseases in Xinjiang Kazakhs and may be of important reference value for the prediction and control of CVD for the Xinjiang area extending to the Belt and Road countries including Kazakhstan.

## Materials and methods

### Study population

The Kazakh residents were from 6 villages in Xinyuan County, Xinjiang Kazakh Autonomous Region. The baseline survey was conducted from April 2009 to December 2013, and was completed in 2,644 permanent residents older than 18 years (1,085 men and 1,559 women). Between April 2016 and August 2017, 2,286 subjects from the baseline population who participated for 5 years or more in the follow-up survey were evaluated for the incidence of CVD; the follow-up rate was 86.46%. Of the them in the baseline population, 706 were first diagnosed with MetS according to the Joint Interim Statement [6], with a prevalence of 30.88% (the age-adjusted prevalence of 28.61%), including 267 men and 439 women. Exclusion criteria at baseline identified 281 subjects with CVD and the remaining 2005 were analyzed. Of 706 subjects with MetS, 191 (82 men and 109 women) had CVD diagnosed by physicians, as case group. Controls (n = 384; men = 164, women = 220) were randomly selected from individuals without MetS or CVD in the case-control study design. These subjects were used for internal verification. Another Kazakh research site, Halabula Township, was the source of 243 external verification controls, selected in August 2017 at the end of the information collection period. Of these, 24 were excluded because of CVD or lack of information at baseline, and 219 (89 men, 130 women) were included for analysis. By the end of the study, CVD developed in 30 of the 219. The cumulative incidence rate was 13.7% (12.4% for men and 14.6% for women).

### Selection and measurement of biomarkers

We extracted 18 kinds of CVD related routine examination index from the MetS metabolic network, including body weight, waist circumference (WC), body adiposity index (BAI), systolic blood pressure (SBP), diastolic blood pressure (DBP), high-density lipoprotein cholesterol (HDL-C), apolipoprotein A (APOA), fasting blood glucose (FBG), fructosamine (FMN), alanine aminotransferase (ALT), aspartate aminotransferase (AST), α-hydroxybutyrate dehydrogenase (α-HBDH), total bilirubin (TBIL), indirect bilirubin (IBIL), albumin (ALB), uric acid (UA), serum creatinine (CREA) and blood urea nitrogen (BUN). Among them, WC, SBP, DBP, HDL-C and FBG were all defined in the traditional MetS. Also, others have been confirmed in the study to meet the above criteria[7–12]. All blood samples were analyzed using an automatic biochemical analyzer (Olympus AU 2700; Olympus Diagnostics, Hamburg, Germany).

### Methods

All participants filled out questionnaires covering demographic data and the history of disease. Physical examination including height, weight, WC, hip circumference and blood pressure, height and weight requirements of wearing light clothes to measure. BAI was calculated as [hip circumference (cm) / (height (m))^1.5^–18]. After each subject was rested for 15 min, each subject measured the blood pressure three times using a mercury sphygmomanometer and then calculated the average value. Each participant signed an informed consent form. The study was approved by the Institutional Ethics Review (IERB) for the First Affiliated Hospital of Shihezi University (IERB No.SHZ2010LL01). Outcome information was obtained directly by examining the medical records of the local hospital discharge records and medical insurance.

### Definition of MetS and CVD

Based on the diagnostic criteria recommended by the JIS[6], MetS was defined as presence of three or more of the following five risk factors: 1) WC≥85 and 80 cm for male and female, respectively; 2) Triglyceride (TG)≥1.70mmol/L; 3) HDL-C<1.00 mmol/L in male and HDL-C<1.30 mmol/L in female; 4) SBP≥130mmHg or DBP≥85mmHg; 5) FPG≥5.6mmol/L.

The following event is identified as an ending event: 1) Hospitalizations for CVD at follow-up; coronary intervention (cardiac catheterization or coronary bypass), angina (or nitroglycerin after cohort study) and CVD death (ICD9: codes 390–495); 2) According to the hospital medical records of hospitalized dued to the following reasons: coronary artery atherosclerosis, coronary heart disease, unstable angina, myocardial infarction, heart failure, stroke, transient cerebral ischemia and peripheral vascular disease (abdominal aortic aneurysm, peripheral vascular surgery or carotid endarterectomy); 3) The CVD events were recorded according to the hospitalization records and questionnaires. If two or more events of the same class of subjects occur, the first occurrence was the outcome event.

### Statistical analysis

#### Descriptive analysis

For patients with MetS, Student's t test (for continuous variables) was used to evaluate significant differences between men and women for 18 biomarkers.

#### Steps of the development of synthetic predictor

EFA with principal component algorithm and varimax rotation from correlation matrix was performed to extract independent factors of MetS from above 18 manifest biomarkers for male and female MetS groups respectively. The criteria for retaining factors were set up as eigenvalue>1 as well as accounting for 75% of the total variation. Only variables that shared at least 15% of the factor variance, corresponding to a factor loading of at least 0.45 were used for further analytical interpretation. After EFA, the clinical significance of each latent factor was named, and Synthetic predictor (SP) [19] was created using a weighted approach: SP = γ_1_F_1_+γ_2_F_2_+… + γ_n_F_n_, where F_1_, F_2_, …Fn were the extracted independent factors with specific clinical significance from the 18 manifest biomarkers, and γ_1_, γ_2_, …γ_n_ denoted their risks to CVD, which were partial regression coefficients in LRD regression models described below.

$$P=\frac{\exp\;\left(B\right)}{1‑\exp\;\left(B\right)}$$ (1)

The logistic regression discrimination (LRD), on the basis of the extreme case-control design, was performed using the model, where P was the probability of CVD. B denoted the discriminant vector estimated by logistic regression, where B = *β*_0_ + *β*_1_*age* + *γ*_1_*F*_1_ + *γ*_2_*F*_2_ ⋯ *γ_n_F_n_* or B = *β*_0_ + *β*_1_*age* + *SP*.

According to the logistic regression discrimination, the results of the internal verification population and the external verifying crowd were evaluated respectively. The probability of each participants with CVD was calculated and the ROC curve was analyzed with the actual CVD follow-up outcome. Calculated the sensitivity and specificity of the disease probability at each cut-off point, and determine the cut-off point with superior sensitivity and specificity.

All statistical analysis for the predictive models was performed using SPSS version 19.0 for Windows. The area under the curve (AUC) for the receiver operating characteristic (ROC) curve analysis, together with the sensitivity, specificity, and cutoff *P* values, were determined using MedCalc software. A two-sided *P* value <0.05 was considered statistically significant.

## Results

In our study, the prevalence of MetS was 30.88% (the standardized prevalence rate was 28.61%) at baseline. 359 CVD events were diagnosed, with a prevalence of 13.87%. Table 1 shows the distribution of age and 18 biomarkers between men and women with MetS. All variables were significantly different between genders. Of these biomarkers, age, weight, WC, SBP, DBP, FBG, FMN, ALT, AST, TBIL, IBIL, ALB, UA, CREA and BUN were higher in men than in women, while BAI, HDL-C, APOA and α-HBDH were higher in women than in men. S1 Table showed the baseline population distribution of age and the eighteen biomarkers between male and female groups, indicating that all variables except FMN, α-HBDH and MS were significantly different between men and women.

**Table 1:** Distribution of age and the eighteen biomarkers between male and female metabolic syndrome groups.

	Male(n = 267)		Female(n = 439)		*P* value
	mean	SD	mean	SD	
Age (years)	50.26	12.80	48.29	12.67	0.046
Weight (kg)	75.72	12.40	65.70	12.32	*P*<0.001
Waistline (cm)	94.20	10.59	89.87	10.74	*P*<0.001
BAI	27.65	3.63	32.19	4.78	*P*<0.001
SBP (mmHg)	144.94	21.43	140.77	26.38	0.030
DBP (mmHg)	92.43	13.66	89.08	15.33	0.004
HDL-C (mmol/L)	1.20	0.41	1.26	0.38	0.042
APOA (g/l)	1.21	0.30	1.24	0.28	0.030
FBG (mmol/L)	6.07	2.14	5.66	1.40	0.002
FMN (umol/l)	228.36	63.79	215.26	36.14	0.001
ALT (IU/L)	21.30	15.27	16.88	13.22	*P*<0.001
AST (IU/L)	33.02	27.56	26.78	15.88	*P*<0.001
α-HBDH (IU/L)	127.52	40.73	134.06	40.45	0.040
TBIL (umol/l)	11.28	6.70	9.28	5.26	*P*<0.001
IBIL (umol/l)	8.55	5.29	6.88	4.09	*P*<0.001
ALB (g/l)	41.72	8.62	39.13	8.84	*P*<0.001
UA (umol/L)	288.77	98.23	213.00	75.64	*P*<0.001
CREA (umol/l)	70.43	13.43	56.93	12.27	*P*<0.001
BUN (mmol/l)	4.89	1.40	4.35	1.21	*P*<0.001

**Note:** BAI: Body adiposity index; SBP: Systolic blood pressure; DBP:Diastolic blood pressure; HDL-C: High-density lipoprotein cholesterol; APOA: Apolipoprotein A; FBG: Fasting blood-glucose; FMN: Fructosamine; ALT: Alanine aminotransferase; AST: Aspartate transferase; α-HBDH: α-Hydroxybutyrate dehydrogenase; TBIL: Total bilirubin; IBIL:Indirect bilirubin; ALB: Serum albumin; UA: Serum uric acid; CREA: Creatinine; BUN: Blood urea nitrogen.

S2 Table shows significant correlations for most biomarkers. In all subjects, weight, WC, SBP, DBP, HDL-C, APOA, ALT, AST, α-HBDH, TBIL, IBIL, ALB, UA, and CREA were significantly correlated with most variables analyzed. The BAI, FBG, FMN and CREA were correlated with approximately half of the variables analyzed. The Kaiser-Meyer-Olkin test values in the male and female groups were 0.636 and 0.632 respectively, and Bartlett's sphericity test for 2 groups had a significance of *P*<0.001. This suggested that the variables were not independent and were strongly correlated, making them suitable for EFA.

Table 2 shows the explained variance, cumulative variance, and loading of the first 7 factors. The results suggested that total variance of 74.02% and 73.74% was explained by the first 7 factors for men and women, respectively. Following the criteria of interpretation mentioned in the statistical analysis section, 7 independent factors with their specific clinical significance were retained and named for 2 groups respectively. For the male group, the first factor was named Obesity factor (OF) and contributed by weight & WC & BAI, following Hepatic function factor (HFF) by TBIL & IBIL, Lipid factor (LF) by HDL-C & APOA & ALB, Renal metabolic factor (RMF) by UA & CREA & BUN, Enzyme metabolism factor (EMF) by ALT & AST & α-HBDH, Blood pressure factor (BPF) by SBP & DBP, and Glucose metabolism factor (GMF) by FBG & FMN. For the female group, the first was named HFF and contributed by TBIL & IBIL & ALB, following OF by weight & WC & BAI, LF by HDL-C & APOA, BPF by SBP & DBP, EMF by ALT & AST & α-HBDH, RMF by UA & CREA & BUN, and GMF by FBG & FMN. Although the order of decline in interpretation of differences between men and women is slightly different, their factor patterns were similar. S3 Table shows the standardized scoring coefficients of each factor for male and female groups.

**Table 2:** Factor loadings by principal component analysis with varimax rotation on 18 routine health check-up biomarkers in MetS patients.

	Male (n = 267)							Female (n = 439)						
	Factor1	Factor2	Factor3	Factor4	Factor5	Factor6	Factor7	Factor1	Factor2	Factor3	Factor4	Factor5	Factor6	Factor7
	OF	HFF	LF	RMF	EMF	BPF	GMF	HFF	OF	LF	BPF	EMF	RMF	GMF
Weight	**0.867**	0.039	-0.099	0.073	0.032	0.060	-0.021	0.071	**0.860**	-0.115	-0.011	0.100	0.010	-0.002
Waistline	**0.918**	0.006	-0.022	0.019	0.077	0.081	0.046	0.054	**0.910**	-0.010	0.053	0.015	-0.021	0.042
BAI	**0.745**	0.025	0.133	-0.011	-0.089	0.165	0.133	-0.055	**0.750**	0.121	0.222	-0.038	0.060	0.007
SBP	0.131	0.021	0.091	0.028	-0.070	**0.894**	0.078	0.039	0.109	0.054	**0.929**	-0.010	0.002	0.044
DBP	0.148	-0.019	0.049	-0.028	0.061	**0.900**	-0.047	0.094	0.118	-0.006	**0.916**	0.050	0.027	-0.003
HDL-C	0.015	0.103	**0.938**	-0.008	0.053	0.073	-0.003	0.287	-0.012	**0.899**	0.022	0.078	-0.022	0.017
APOA	-0.055	0.077	**0.926**	0.051	0.089	0.110	0.011	0.109	-0.018	**0.906**	0.012	-0.003	0.078	0.075
FBG	0.115	-0.041	0.026	-0.166	0.055	0.046	**0.806**	-0.008	0.061	0.006	0.016	-0.023	0.048	**0.920**
FMN	0.014	0.206	0.001	0.360	0.155	-0.027	**0.596**	0.195	-0.040	0.199	0.036	0.441	0.045	**0.649**
ALT	0.149	0.067	0.014	-0.011	**0.806**	-0.090	0.085	0.117	0.052	-0.004	-0.063	**0.749**	-0.014	0.113
AST	0.033	0.184	-0.008	-0.015	**0.801**	-0.005	0.019	0.266	0.051	-0.064	-0.037	**0.732**	-0.055	0.093
α-HBDH	-0.170	-0.011	0.170	0.068	**0.569**	0.085	0.067	-0.117	-0.021	0.229	0.207	**0.621**	0.185	-0.088
TBIL	-0.013	**0.957**	0.101	0.066	0.113	0.032	0.053	**0.918**	0.005	0.119	0.056	0.171	0.019	0.038
IBIL	0.030	**0.957**	0.077	0.002	0.083	0.003	0.024	**0.912**	-0.002	0.187	0.045	0.033	0.024	-0.007
ALB	0.178	0.440	**0.468**	0.366	0.172	-0.139	0.166	**0.537**	0.096	0.432	0.092	0.203	0.041	0.150
UA	0.187	0.384	0.274	**0.525**	0.299	-0.056	-0.064	0.408	0.302	0.020	0.013	0.120	**0.530**	0.184
CREA	-0.026	0.118	-0.149	**0.806**	0.041	0.022	-0.153	-0.009	-0.002	-0.127	-0.035	-0.078	**0.853**	-0.013
BUN	0.029	-0.122	0.172	**0.786**	-0.112	0.013	0.176	-0.011	-0.039	0.187	0.060	0.093	**0.729**	0.028
% Variance explained	20.450	13.677	10.400	9.000	7.764	7.045	5.687	21.316	13.317	9.397	8.501	8.198	7.040	5.972
Cumulative variance	20.450	34.128	44.528	53.528	61.292	68.338	**74.025**	21.316	34.633	44.030	52.532	60.730	67.770	**73.742**

**Note:** Factors were named as Obesity factor(OF),Hepatic function factor (HFF), Lipid factor (LF), Enzyme metabolic factor(EMF),Blood pressure factor (BPF), Renal metabolic factor(RMF), Glucose metabolism factor(GMF). **Bold** indicates that the absolute value of the factor loading was >0.45.

**Fig 1:**
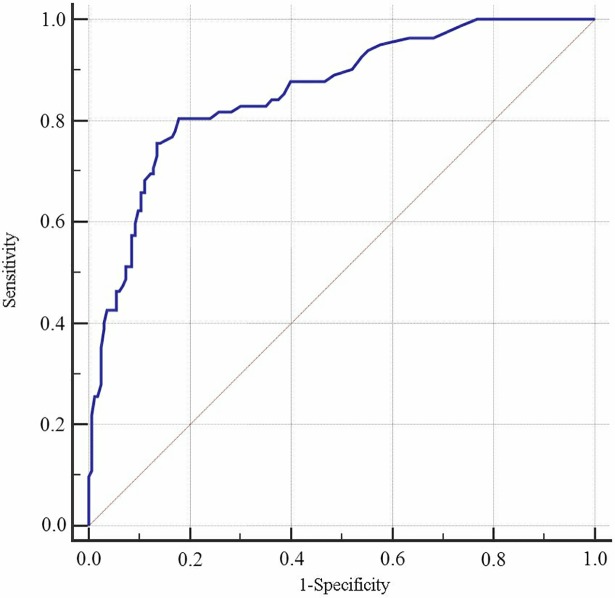
ROC curve for discrimination and prediction of CVD. This shows the discriminative curve under internal validation for male.

**Fig 2:**
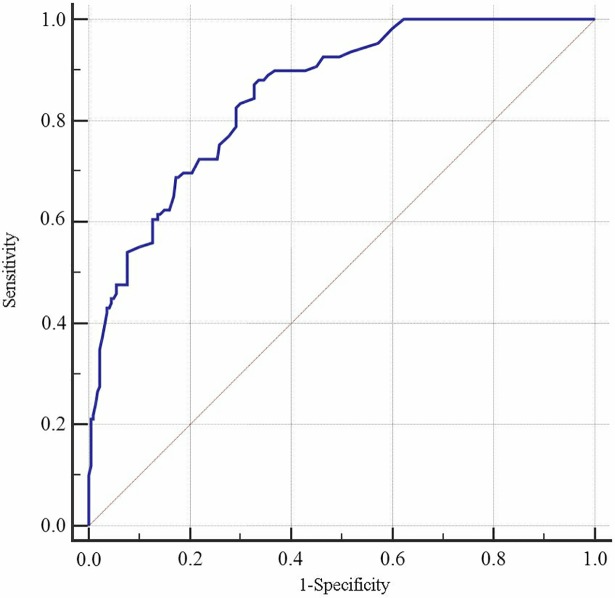
ROC curve for discrimination and prediction of CVD. This shows the discriminative curve under internal validation for female.

**Fig 3:**
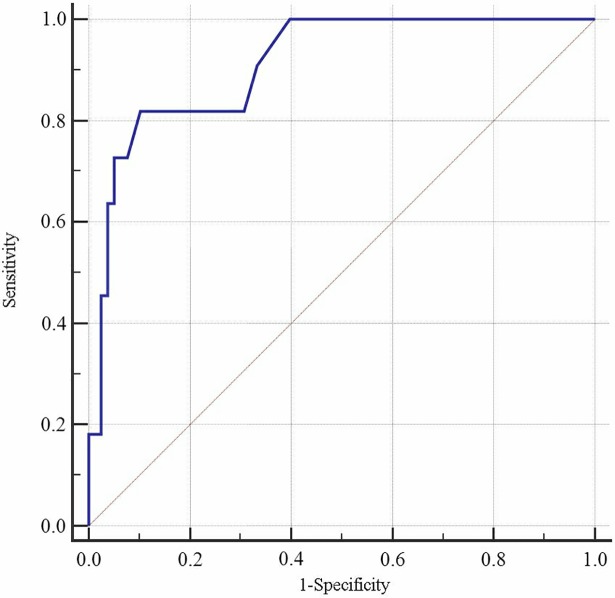
ROC curve for discrimination and prediction of CVD. This shows the discriminative curve under external validation for male.

**Fig 4:**
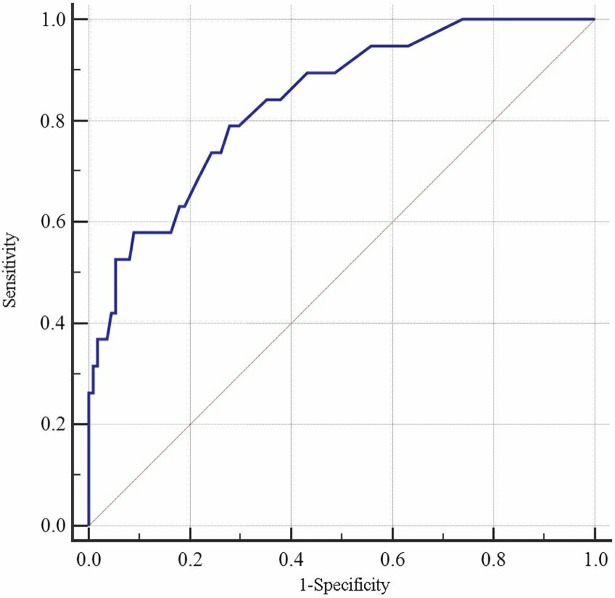
ROC curve for discrimination and prediction of CVD. This shows the discriminative curve under external validation for female.

Figs 1 and 2 show the AUC with sensitivity, specificity, and the cut-off points of *P* values (criterion) for the LRD model from the internal validation design using age and SP as discriminant factors (for men: B = 0.733–0.039Age + SP; for women: B = 0.077–0.016Age + SP). In males, the AUC was 0.857 (95% confidence interval [CI]: 0.807–0.898), with a Youden's index value of 0.622 (Sen = 80.49%, Spe = 81.71%), and an optimal cut-off of 0.30. In females, the AUC was 0.852 (95% CI: 0.809–0.889), with a Youden's index value of 0.544 (Sen = 88.07%, Spe = 66.36%), and an optimal cut-off of 0.23. Figs 3 and 4 show the AUC with sensitivity, specificity, and cut-off points of *P* value (criterion) by LRD model from the external validation design using age and SP as discriminant factors (for men: B = -7.55 + 0.079Age + SP; for women: B = -5.104 + 0.051Age + SP). In males, the AUC was 0.914 (95% CI: 0.832–0.963), with a Youden's index value of 0.0.703 (Sen = 81.82%, Spe = 88.48%), and an optimal cut-off of 0.16. In females, the AUC was 0.848 (95% CI: 0.774–0.905), with a Youden's index value of 0.552 (Sen = 89.47%, Spe = 65.77%), and an optimal cut-off of 0.09. The predictive effect of the CVD model built by SP and age was better, and the predictive ability for CVD was better in Kazakhs men (Table 3 and Table 4).

**Table 3:** Results of logistic regression discrimination for internal and external validation in males and females.

	Model 1			Model 2		
Variables	B	P-value	OR(95%)	B	P-value	OR(95%)
Male						
Constant	0.733	0.442	2.081	-7.550	P<0.001	0.001
Age	-0.039	0.038	0.962(0.928–0.998)	0.079	0.019	1.082(1.013–1.156)
OF	1.561	P<0.001	4.765(2.839–7.996)	0.683	0.101	1.980(0.875–4.479)
HFF	-0.244	0.168	0.784(0.554–1.108)	0.555	0.047	1.742(1.007–3.014)
LF	-0.597	0.004	0.551(0.367–0.826)	-0.576	0.268	0.562(0.203–1.557)
RMF	0.259	0.190	1.295(0.880–1.906)	0.167	0.740	1.182(0.441–3.170)
EMF	-0.372	0.101	0.690(0.442–1.075)	0.363	0.415	1.438(0.601–3.442)
BPF	0.636	0.003	1.888(1.247–2.860)	0.549	0.172	1.732(0.788–3.808)
GMF	0.624	0.002	1.867(1.247–2.795)	-0.051	0.912	0.950(0.383–2.355)
Female						
Constant	0.077	0.916	1.08	-5.104	0.002	0.006
Age	-0.016	0.260	0.984(0.956–1.012)	0.051	0.059	1.053(0.998–1.111)
HFF	-0.511	0.003	0.600(0.427–0.843)	0.049	0.895	1.051(0.504–2.191)
OF	1.168	P<0.001	3.217(2.248–4.602)	0.972	0.008	2.642(1.291–5.409)
LF	-0.61	P<0.001	0.543(0.387–0.763)	-0.220	0.539	0.802(0.397–1.621)
BPF	0.693	P<0.001	1.999(1.399–2.858)	0.674	0.036	1.961(1.044–3.685)
EMF	-0.279	0.103	0.756(0.541–1.058)	0.125	0.680	1.133(0.626–2.052)
RMF	0.472	0.009	1.603(1.124–2.287)	-0.325	0.427	0.723(0.325–1.609)
GMF	0.892	P<0.001	2.440(1.610–3.698)	0.583	0.186	1.791(0.755–4.246)

**Note: Model 1**, Internal validation. For male: B = 0.733–0.039Age + SP, SP = 1.561OF—0.244HFF—0.597LF + 0.259RMF—0.372EMF + 0.636BPF + 0.624GMF; for female: B = 0.077–0.016Age+SP, SP = -0.511HFF + 1.168OF—0.610LF + 0.693BPF—0.279EMF +0.472RMF + 0.892GMF. **Model 2**, External validation. For male: B = - 7.55 + 0.079Age + SP, SP = 0.101OF + 0.555HFF—0.576LF + 0.167RMF + 0.363EMF + 0.549BPF—0.051GMF; for female: B = -5.104+0.051Age+SP, SP = 0.049HFF + 0.972OF—0.220LF + 0.674BPF + 0.125EMF—0.325RMF + 0.583GMF.

**Table 4:** Results of ROC curves for internal and external validation in males and females.

	Group	Cut-off	Sen(%)	Spe(%)	Youden's index	AUC(95%CI)	*P*-value
Model 1	Male	0.30	80.49	81.71	0.622	0.857(0.807–0.898)	*P*<0.001
	Female	0.23	88.07	66.36	0.544	0.852(0.809–0.889)	*P*<0.001
Model 2	Male	0.16	81.82	88.46	0.703	0.914(0.832–0.963)	*P*<0.001
	Female	0.09	89.47	65.77	0.552	0.848(0.774–0.905)	*P*<0.001

**Note:** Model 1, Internal validation; Model 2, External validation. Abbreviation: Sen-sensitivity; Spe-specificity.

## Discussion

The standardized prevalence rate of MetS in Kazakhs was 28.61%, which was much higher than the national MetS prevalence rate of 16.5% [24]. Studies have shown that individuals with MetS are more likely to develop CVD [25,26], and a higher prevalence of MetS suggests that the risk of developing CVD in the Kazakh population may be higher.

Based on the MetS multi-etiology hypothesis [27], this study used EFA to extract 7 principal components according to sex from18 risk factors that represent 7 potential factors for MetS occurrence in Kazakhs. Of the 7 potential factors, the first 3 factors for men and women were OF (WC & weight & BAI), HFF (TBIL & IBIL), and LF (HDL-C & APOA). Among these, obesity and dyslipidemia are classic components of MetS [28,29]. Body mass index (BMI) and WC are commonly used as indicators of obesity, and some studies have suggested that body fat content is a major risk factor for MetS, because the BAI is better than the BMI in estimating body fat content [30,31]. In this study, weight, WC, and BAI were used as the obesity factors, providing more comprehensive results than the W Zhang [32] study using BMI and WC as the obesity factors. The main characteristic of blood lipids in this study was the high prevalence of decreased levels of HDL-C [33]. Therefore, we extracted HDL-C and APOA as blood lipid factors in MetS, and the contribution of 2 lipid variables was greater than the contribution of the 4 variables reported in a previous study [34], indicating that the lipids extracted in this study are more accurate, and that these factors can better reflect the lipid accumulation characteristics in Kazakhs with MetS. In Kazakh MetS patients with HFF as the dominant factor, male sex as the second factor, and female sex as the first factor, the results are different from those in the Han Chinese population [34]. HFF may be an important factor in the assessment of MetS in Kazakhs. The contribution of EMT (ALT & AST & α-HBDH) was not high in our study, but was an important indicator for the diagnosis of non-alcoholic fatty liver disease. Non-alcoholic fatty liver disease has been shown to increase the risk of developing MetS, and the prevalence is higher in Kazakhs than in local Han Chinese [35, 36]. Therefore, ALT, AST and α-HBDH were selected as Kazakh MetS liver enzyme factors. In our study, the contribution of RMF (UA & CREA & BUN) was higher in men (9.00%) than in women (7.04%). In Wen-chao Zhang's study [15], RMF was not extracted for men, but the female RMF contribution rate (6.81%) was similar to the rate in this study. It may be due to the reason that this was not included in CREA, leading to a weak RMF aggregation in men. Blood pressure and glucose metabolism in this study were male’s 6th and 7th factors, and female’s 4th and 7th factors, both contributing less than 10%. Consistent with the findings of A Ghosh [37], the contribution of blood pressure and glycemic factors was least. If a study subject was diabetic [38, 39], blood pressure and blood glucose were 1st and 2nd factors, suggesting that due to differences in study populations, the contribution of each factor to MetS was also different. Albumin is a common biochemical indicator and fluctuates in the human body due to disease. In this study, albumin was incorporated into male LF and female HFF, respectively. However, it was highly correlated with the routine physical examination indexes included in this study, and is retained for the accuracy of factor analysis.

In this study, a CVD predictive model was constructed using age and SP as predictors in the Kazakh population, with a predictive ability higher than that of the FRS (AUC approximately 0.80) [40]. This was also higher than the predictive ability achieved while using a Chinese evaluation method for 10-year risk of ischemic CVD (hereinafter referred to as the “Chinese evaluation method”) in the optimal model (for men: AUC = 0.796; for women: AUC = 0.791) and the simple model (for men: AUC = 0.792; for women: AUC = 0.783) [41]. The Chinese evaluation method only used 8 risk factors as model predictors, which is the same as the number of predictive factors in our study. However, the potential factors in this study include obesity, blood pressure abnormalities, dyslipidemia, and glucose metabolism disorders. These cover the risk factors for CVD more comprehensively, and enhance the predictive ability of the model. Li Jiqing's findings showed that the male AUC in training samples was lower than that in the current study (0.837 vs 0.857) [16]. However, the AUC in females was better than the AUC in this study (0.897 vs 0.852). Although the study still used the Chinese evaluation method, it also incorporated electrocardiographic information that was highly correlated with CVD. These data directly reflect CVD status, and can significantly improve the predictive and diagnostic capabilities of the model. Using MetS as a predictor of CVD, the current study only includes the classic components [42, 43], and overlooks the metabolic network of liver enzymes, renal function, bilirubin, and other components that affect CVD. However, Zhenxin Zhu's study [20] incorporated 8 factors of the MetS network into a CVD prediction model, with an AUC as high as 0.994 and 0.998 for men and women. We speculate that the present predictive model can fully cover the risk factors of CVD, which is key to improvement of the predictive ability. Therefore, predictors should cover both the risk factors and the incidence of CVD.

External verification is important for the assessment of the feasibility of the CVD model. The classic FRS model predicts a large predictive error for Hispanic, Asian, and elderly American populations, and significantly overestimates risk in a Han Chinese population [44], but has a better predictive ability for the Mongolian population [45]. Wang Fang's study of a Chinese health check-up group using the Chinese external validation method obtained an AUC of 0.717, which was significantly lower than the AUC in the optimal model and simple model [46]. It may be that because the outcome measured was carotid atherosclerotic plaque, the perdictive power was reduced. Using external verification to validate the established model, the AUC of men was 0.914 (95% CI: 0.832–0.963) and the AUC of women was 0.848 (95% CI: 0.774–0.905), both of which were greater than or equal to 0.85. This shows that the CVD model can be applied to local Kazakhs, especially for male Kazakh CVD prediction. Based on age and SP, this study can be used to predict the incidence of CVD in the local Kazakh population, which is conducive to early screening in primary health institutions, and can thus help reduce the overall incidence of CVD by controlling risk factors.

Our study had the following advantages. First, the study is based on the characteristics of local Kazakh disease. The prevalence rates of MetS, hypertension, and obesity in Kazakhs are significantly obviously higher than those in other ethnic groups residing in the same area. MetS was used to construct a CVD prediction model for the Kazakh population, as similar studies have not been reported. Second, the results of this study are based on indicators used in routine physical examinations. The indicators are all derived from national health checkup projects supported by the Chinese government. These indicators are easy to determine and are cost effective. Third, this study used EFA to extract 7 potential factors from 18 routine physical examination indicators to form a SP, and to a greater extent to strengthen the predictive ability of the model. This was not only a simple summation of factors, but also showed a correlation between CVD prediction results and potential factors. Finally, the model underwent internal and external validations to illustrate the applicability. Therefore, this model can be applied to local Kazakh residents for early prevention of CVD and for screening of high-risk individuals.

This study had some limitations. First, routine physical examination indicators cannot cover all risk factors for CVD, resulting in limited predictive ability for the model. Second, the sample size is not big enough. Since the Kazakh lifestyle is mainly nomadic, data were not collected for CVD patients who did not go to the hospital or those whose hospitalization records were missing. Therefore, this study may underestimate the cumulative incidence of CVD. Subsequent research can supplement follow-up information, increase the number of routine physical examination indicators, extract potential factors that can cover more comprehensive information, and improve the predictive ability of the model.

## Supporting information

S1 Table Distribution of age and the eighteen biomarkers between male and female groups. (DOCX)

S2 Table Correlation matrix of eighteen biomarkers. (DOC)

S3 Table The standardized scoring coefficients of each factor for males and females. (DOCX)
