# Correction: Metabolic syndrome in Xinjiang Kazakhs and construction of a risk prediction model for cardiovascular disease risk

**DOI:** 10.1371/journal.pone.0210907

**Published:** 2019-01-10

**Authors:** Lei Mao, Jia He, Xiang Gao, Heng Guo, Kui Wang, Xianghui Zhang, Wenwen Yang, Jingyu Zhang, Shugang Li, Yunhua Hu, Lati Mu, Yizhong Yan, Jiaolong Ma, Yusong Ding, Mei Zhang, Jiaming Liu, Rulin Ma, Shuxia Guo

In the Materials and Methods section, there is an error in the first equation in the section titled “Steps of the development of synthetic predictor”. The subtraction sign in the denominator is incorrect. It should be an addition sign. Please view the complete, correct equation here:

$$P=\frac{exp(B)}{1+exp(B)}$$
