# Supplementary material for: Metabolic syndrome in Xinjiang Kazakhs and construction of a risk prediction model for cardiovascular disease risk
Source: PLoS One. 2018 Sep 6;13(9):e0202665. doi: 10.1371/journal.pone.0202665 (PMC6126809; doi:10.1371/journal.pone.0202665)
Supplement: S1 Table — (DOCX) [file pone.0202665.s001.docx]

**Table S1. Distribution of age and the eighteen biomarkers between male and female groups.**

|  | Male(n=908) | | Female(n=1378) | | *P* value |
| --- | --- | --- | --- | --- | --- |
|  | mean | SD | mean | SD |  |
| Age(years) | 43.83 | 13.78 | 41.72 | 12.82 | *P*<0.001 |
| Weight(kg) | 68.55 | 11.84 | \9.30 | 11.19 | *P*<0.001 |
| Waistline(cm) | 85.60 | 11.15 | 81.71 | 11.26 | *P*<0.001 |
| BAI | 25.67 | 3.64 | 29.77 | 4.63 | *P*<0.001 |
| SBP(mmHg) | 133.90 | 23.12 | 128.27 | 23.42 | *P*<0.001 |
| DBP(mmHg) | 85.13 | 14.36 | 81.81 | 14.53 | *P*<0.001 |
| HDL-C(mmol/L) | 1.31 | 0.62 | 1.38 | 0.40 | 0.002 |
| APOA(g/l) | 1.23 | 0.28 | 1.29 | 0.30 | *P*<0.001 |
| FBG(mmol/L) | 5.38 | 1.49 | 5.17 | 1.13 | *P*<0.001 |
| FMN(umol/l) | 188.58 | 79.92 | 184.70 | 96.08 | 0.296 |
| ALT(IU/L) | 18.23 | 15.51 | 15.21 | 11.50 | *P*<0.001 |
| AST(IU/L) | 30.41 | 19.87 | 26.64 | 19.56 | *P*<0.001 |
| α-HBDH(IU/L) | 130.42 | 45.73 | 131.84 | 45.39 | 0.463 |
| TBIL(umol/l) | 11.42 | 5.92 | 9.73 | 4.88 | *P*<0.001 |
| DBIL(umol/l) | 8.73 | 4.68 | 7.36 | 3.90 | *P*<0.001 |
| ALB(g/l) | 41.52 | 8.37 | 40.14 | 8.52 | *P*<0.001 |
| UA(umol/L) | 271.99 | 86.24 | 202.13 | 74.12 | *P*<0.001 |
| CREA(umol/l) | 69.90 | 18.56 | 55.63 | 11.49 | *P*<0.001 |
| BUN(mmol/l) | 4.80 | 1.54 | 4.17 | 1.13 | *P*<0.001 |
| MS(n , %) ^#^ | 267 (29.4%) | | 439 (31.9%) | | 0.214 |

**Note:** BAI: Body adiposity index; SBP: Systolic blood pressure; DBP:Diastolic blood pressure; HDL-C: High-density lipoprotein cholesterol; APOA: Apolipoprotein A; FBG: Fasting blood-glucose; FMN: Fructosamine; ALT: Alanine aminotransferase; AST: Aspartate transferase; α-HBDH: α-Hydroxybutyrate dehydrogenase; TBIL: Total bilirubin; DBIL:Indirect bilirubin; ALB: Serum albumin; UA: Serum uric acid; CREA: Creatinine; BUN: Blood urea nitrogen; MS: metabolic syndrome. ^#^: Descriptive characteristics were compared by the χ^2^ test.
