# Supplementary material for: Metabolic syndrome in Xinjiang Kazakhs and construction of a risk prediction model for cardiovascular disease risk
Source: PLoS One. 2018 Sep 6;13(9):e0202665. doi: 10.1371/journal.pone.0202665 (PMC6126809; doi:10.1371/journal.pone.0202665)
Supplement: S2 Table — (DOC) [file pone.0202665.s002.doc]

**Table S2. Correlation matrix of eighteen biomarkers.**

|  | Weight (kg) | Waistline (cm) | BAI | SBP (mmHg) | DBP (mmHg) | HDL-C (mmol/L) | APOA (g/l) | FBG (mmol/L) | FMN (umol/l) | ALT (IU/L) | AST (IU/L) | α-HBDH (IU/L) | TBIL (umol/l) | DBIL (umol/l) | ALB (g/l) | UA (umol/L) | CREA (umol/l) | BUN (mmol/l) |
| --- | --- | --- | --- | --- | --- | --- | --- | --- | --- | --- | --- | --- | --- | --- | --- | --- | --- | --- |
| Weight(kg) | 1 | 0.748^a^ | 0.200^a^ | 0.122^a^ | 0.198^a^ | -0.087^b^ | -0.102^a^ | 0.098^a^ | 0.092^a^ | 0.158^a^ | 0.119^a^ | -0.054 | 0.096^a^ | 0.114^a^ | 0.164^a^ | 0.324^a^ | 0.191^a^ | 0.082^b^ |
| Waistline(cm) |  | 1 | 0.435^a^ | 0.176^a^ | 0.191^a^ | -0.004 | -0.045 | 0.122^a^ | 0.082^b^ | 0.117^a^ | 0.104^a^ | -0.022 | 0.085^b^ | 0.094^a^ | 0.132^a^ | 0.250^a^ | 0.073^b^ | 0.063^b^ |
| BAI |  |  | 1 | 0.205^a^ | 0.141^a^ | 0.106^a^ | 0.075^b^ | 0.028 | -0.031 | -0.055 | -0.073^b^ | 0.049 | -0.042 | -0.052 | 0.020 | -0.056 | -0.185^a^ | -0.036 |
| SBP(mmHg) |  |  |  | 1 | 0.744^a^ | 0.103^a^ | 0.098^a^ | 0.076^b^ | 0.047 | -0.032 | -0.002 | 0.079^b^ | 0.076^b^ | 0.061 | 0.096^a^ | 0.099^a^ | 0.020 | 0.068^b^ |
| DBP(mmHg) |  |  |  |  | 1 | 0.072^b^ | 0.075^b^ | 0.036 | 0.032 | 0.041 | 0.045 | 0.098^a^ | 0.094^a^ | 0.076^b^ | 0.095^a^ | 0.094^a^ | 0.045 | 0.057 |
| HDL-C(mmol/L) |  |  |  |  |  | 1 | 0.826^a^ | 0.030 | 0.122^a^ | 0.070^b^ | 0.106 | 0.139^a^ | 0.276^a^ | 0.301^a^ | 0.460^a^ | 0.128^a^ | -0.109^a^ | 0.141^a^ |
| APOA(g/l) |  |  |  |  |  |  | 1 | 0.044 | 0.139^a^ | 0.07^b^ | 0.040 | 0.178^a^ | 0.20^1a^ | 0.204^a^ | 0.349^a^ | 0.184^a^ | -0.043 | 0.128^a^ |
| FBG(mmol/L) |  |  |  |  |  |  |  | 1 | 0.226^a^ | 0.141^a^ | 0.105^a^ | -0.008 | 0.039 | 0.047 | 0.102^a^ | 0.113^a^ | 0.027 | 0.048 |
| FMN(umol/l) |  |  |  |  |  |  |  |  | 1 | 0.230^a^ | 0.238^a^ | 0.175^a^ | 0.294^a^ | 0.206^a^ | 0.343^a^ | 0.243^a^ | 0.101^a^ | 0.221^a^ |
| ALT(IU/L) |  |  |  |  |  |  |  |  |  | 1 | 0.470^a^ | 0.235^a^ | 0.224^a^ | 0.174^a^ | 0.213^a^ | 0.250^a^ | 0.061 | 0.041 |
| AST(IU/L) |  |  |  |  |  |  |  |  |  |  | 1 | 0.200^a^ | 0.295^a^ | 0.237^a^ | 0.208^a^ | 0.284^a^ | 0.077^b^ | 0.012 |
| α-HBDH(IU/L) |  |  |  |  |  |  |  |  |  |  |  | 1 | 0.100^a^ | 0.068^b^ | 0.162^a^ | 0.079b | -0.012 | 0.072^b^ |
| TBIL(umol/l) |  |  |  |  |  |  |  |  |  |  |  |  | 1 | 0.917^a^ | 0.477^a^ | 0.399^a^ | 0.126^a^ | 0.076^b^ |
| DBIL(umol/l) |  |  |  |  |  |  |  |  |  |  |  |  |  | 1 | 0.451^a^ | 0.359^a^ | 0.122^a^ | 0.066^b^ |
| ALB(g/l) |  |  |  |  |  |  |  |  |  |  |  |  |  |  | 1 | 0.463^a^ | 0.099^a^ | 0.191^a^ |
| UA(umol/L) |  |  |  |  |  |  |  |  |  |  |  |  |  |  |  | 1 | 0.466^a^ | 0.291^a^ |
| CREA(umol/l) |  |  |  |  |  |  |  |  |  |  |  |  |  |  |  |  | 1 | 0.444^a^ |
| BUN(mmol/l) |  |  |  |  |  |  |  |  |  |  |  |  |  |  |  |  |  | 1 |

**Note:** a Significant correlation, P<0.01; b Significant correlation, P<0.05. BAI: Body adiposity index; SBP: Systolic blood pressure; DBP:Diastolic blood pressure; HDL-C: High-density lipoprotein cholesterol; APOA: Apolipoprotein A; FBG: Fasting blood-glucose; FMN: Fructosamine; ALT: Alanine aminotransferase; AST: Aspartate transferase; α-HBDH: α-Hydroxybutyrate dehydrogenase; TBIL: Total bilirubin; DBIL:Indirect bilirubin; ALB: Serum albumin; UA: Serum uric acid; CREA: Creatinine; BUN: Blood urea nitrogen.
