# Supplementary material for: Metabolic syndrome in Xinjiang Kazakhs and construction of a risk prediction model for cardiovascular disease risk
Source: PLoS One. 2018 Sep 6;13(9):e0202665. doi: 10.1371/journal.pone.0202665 (PMC6126809; doi:10.1371/journal.pone.0202665)
Supplement: S3 Table — (DOCX) [file pone.0202665.s003.docx]

**Table S3 The standardized scoring coefficients of each factor for males and females.**

|  | **Male (n=267)** | | | | | | | **Female (n=439)** | | | | | | |
| --- | --- | --- | --- | --- | --- | --- | --- | --- | --- | --- | --- | --- | --- | --- |
|  | Factor1 | Factor2 | Factor3 | Factor4 | Factor5 | Factor6 | Factor7 | Factor1 | Factor2 | Factor3 | Factor4 | Factor5 | Factor6 | Factor7 |
|  | OF | HFF | LF | RMF | EMF | BPF | GMF | HFF | OF | LF | BPF | EMF | RMF | GMF |
| Weight | 0.395 | -0.006 | -0.049 | 0.012 | 0.001 | -0.055 | -0.098 | 0.007 | 0.396 | -0.046 | -0.091 | 0.044 | -0.024 | -0.046 |
| Waistline | 0.414 | -0.035 | -0.008 | -0.026 | 0.024 | -0.057 | -0.045 | -0.015 | 0.420 | 0.020 | -0.062 | -0.021 | -0.050 | -0.001 |
| BAI | 0.323 | -0.010 | 0.068 | -0.046 | -0.091 | -0.003 | 0.059 | -0.089 | 0.337 | 0.103 | 0.047 | -0.041 | 0.007 | -0.021 |
| SBP | -0.054 | 0.027 | -0.031 | 0.034 | -0.028 | 0.541 | 0.058 | -0.013 | -0.044 | -0.023 | 0.527 | -0.038 | -0.025 | 0.032 |
| DBP | -0.036 | 0.000 | -0.047 | 0.009 | 0.074 | 0.549 | -0.067 | 0.021 | -0.044 | -0.069 | 0.519 | 0.003 | -0.008 | -0.017 |
| HDL-C | 0.014 | -0.051 | 0.474 | -0.081 | -0.034 | -0.033 | -0.057 | -0.026 | 0.018 | 0.473 | -0.041 | -0.025 | -0.052 | -0.061 |
| APOA | -0.027 | -0.071 | 0.459 | -0.039 | -0.007 | 0.003 | -0.042 | -0.119 | 0.021 | 0.508 | -0.044 | -0.063 | 0.017 | 0.003 |
| FBG | -0.015 | -0.037 | -0.014 | -0.124 | -0.031 | 0.004 | 0.743 | -0.056 | -0.006 | -0.044 | 0.006 | -0.158 | -0.021 | 0.750 |
| FMN | -0.068 | 0.036 | -0.083 | 0.170 | 0.006 | 0.003 | 0.527 | -0.031 | -0.055 | 0.025 | 0.005 | 0.155 | -0.026 | 0.440 |
| ALT | 0.052 | -0.087 | -0.040 | -0.043 | 0.479 | -0.045 | -0.007 | -0.051 | 0.008 | -0.052 | -0.055 | 0.460 | -0.035 | -0.031 |
| AST | -0.011 | -0.014 | -0.070 | -0.045 | 0.473 | 0.029 | -0.060 | 0.047 | -0.002 | -0.114 | -0.039 | 0.433 | -0.064 | -0.047 |
| α-HBDH | -0.109 | -0.104 | 0.038 | 0.026 | 0.347 | 0.084 | 0.017 | -0.203 | -0.030 | 0.123 | 0.099 | 0.425 | 0.106 | -0.182 |
| TBIL | -0.048 | 0.472 | -0.065 | -0.066 | -0.074 | 0.046 | 0.011 | 0.452 | -0.045 | -0.117 | 0.005 | -0.032 | -0.028 | -0.052 |
| DBIL | -0.019 | 0.484 | -0.067 | -0.103 | -0.090 | 0.021 | -0.014 | 0.459 | -0.040 | -0.068 | -0.003 | -0.117 | -0.021 | -0.071 |
| ALB | 0.062 | 0.108 | 0.171 | 0.119 | -0.014 | -0.119 | 0.081 | 0.167 | 0.020 | 0.136 | 0.006 | 0.013 | -0.021 | 0.034 |
| UA | 0.065 | 0.074 | 0.060 | 0.238 | 0.105 | -0.040 | -0.140 | 0.161 | 0.096 | -0.083 | -0.044 | -0.026 | 0.306 | 0.069 |
| CREA | -0.043 | -0.003 | -0.146 | 0.481 | 0.016 | 0.065 | -0.164 | 0.004 | -0.030 | -0.093 | -0.029 | -0.061 | 0.559 | -0.037 |
| BUN | -0.026 | -0.150 | 0.045 | 0.462 | -0.096 | 0.010 | 0.144 | -0.080 | -0.043 | 0.082 | 0.013 | 0.036 | 0.463 | -0.039 |

**Note:** BAI: Body adiposity index; SBP: Systolic blood pressure; DBP:Diastolic blood pressure; HDL-C: High-density lipoprotein cholesterol; APOA: Apolipoprotein A; FBG: Fasting blood-glucose; FMN: Fructosamine; ALT: Alanine aminotransferase; AST: Aspartate transferase; α-HBDH: α-Hydroxybutyrate dehydrogenase; TBIL: Total bilirubin; IBIL:Indirect bilirubin; ALB: Serum albumin; UA: Serum uric acid; CREA: Creatinine; BUN: Blood urea nitrogen. Factors were named as Obesity factor(OF),Hepatic function factor (HFF), Lipid factor (LF), Enzyme metabolic factor(EMF),Blood pressure factor (BPF), Renal metabolic factor(RMF), Glucose metabolism factor(GMF).
